# Synthesis of Betaine Copolymer for Surface Modification of Cotton Fabric by Enhancing Temperature-Sensitive and Anti-Protein Specific Absorption Performance

**DOI:** 10.3390/ma14226793

**Published:** 2021-11-11

**Authors:** Xiaofei Yan, Chenkai Zhu, Ju Huang, Dongmin Qi, Jiawei Li

**Affiliations:** 1 Key Laboratory of Advanced Textile Materials and Manufacturing Technology, Ministry of Education, Zhejiang Sci-Tech University, Hangzhou 310018, China; yanxf@zstu.edu.cn; 2 Zhejiang Provincial Key Laboratory of Fiber Materials and Manufacturing Technology, Zhejiang Sci-Tech University, Hangzhou 310018, China; 3 College of Textile Science and Engineering (International Institute of Silk), Zhejiang Sci-Tech University, Hangzhou 310018, China; huangju273@mail.ustc.edu.cn; 4 Ningbo Institute of Technology, Beihang University, Ningbo 315800, China; chenkaizhu@zstu.edu.cn

**Keywords:** betaine copolymer, cotton fabric, temperature-sensitive response, anti-protein specific adhesion

## Abstract

The growth and reproduction of microorganisms on fabrics could not only affect the wearability of textiles but also cause harm to human health, and it is an important problem that should be solved to reduce the adsorption and growth of microorganisms on the surface of the fabric. A series of ω-vinyl betaine copolymers were synthesized by catalytic chain transfer polymerization (CCTP) and were modified by mercapto-vinyl click chemistry to synthesize silane-modified betaine copolymers, which were used to treat the cotton fabric. The hydrophilic–hydrophobic transition performance and anti-protein specific adhesion performance of cotton fabric with the betaine copolymer were systematically investigated. The copolymer was confirmed to be successfully finished on the cotton fabric via ^1^H–NMR and FTIR. The cotton fabric, which was treated by the betaine copolymer, presented temperature response performance in the range of 30–55 °C and had excellent anti-protein adsorption performance. The treated fabric had the best temperature-sensitive and anti-protein specific absorption performance among all the specimens when the mass fraction of G06B in DMAPS was 6 wt.%.

## 1. Introduction

Microbial pathogens are ubiquitous in daily life, which seriously threaten human health [1]. Fabric is the front line of defense for the human body, so extensive research on antibiological fabrics has been carried out in recent years [2]. The main effective solutions to prepare antibacterial fabrics are the introduction of bactericidal groups attached to the surface of the fabric, and the deposition of antibacterial agents (heavy metals or microcapsules) [3,4] on the fabric surface to release bacterial toxic substances. However, the above methods not only have a high cost, low mechanical strength, and poor durability, but may also have toxic effects on humans. Thus, the construction of an antibacterial layer as a physical barrier between the material and biological contaminants has been considered a novel solution to prevent the adhesion of bacteria and reduce the health risk [5].

Currently, attention has been given to the betaine polymer [6,7] in the field of antibacterial materials due to its stable molecular design, nontoxicity, and nonirritation [8,9]. The betaine polymer is a type of zwitterionic polymer [10,11] that contains the same number of opposite charges in the structure [12], and it presents unique physicochemical properties, such as unique temperature sensitivity [13] and suitable high critical dissolution, resulting in an impressive comfortability-adjusting performance [14,15]. The hydration layer formed by the zwitterionic polymer [16] through the strong hydration of the ions is better than that of the PEG polymer, and it has a better contaminant adhesion performance [17,18,19,20]. However, the surface of the betaine polymer against specific protein adsorption performance could be significantly affected with the cumulation of killed bacterial and other biological contaminants gradually [21].

According to the literature [22], fluorinated acrylate polymer has outstanding weather resistance, low friction, and low surface energy, and it has excellent resistance to oxidation, acids, and alkalis, respectively [23]. It is an indispensable functional material in many demanding applications. The fluorine element content and fluorine-containing structure in the fluorine-containing acrylic polymer are the two main factors that affect the performance of the fluorine-containing acrylic polymer [24]. It can form strong polar C–F bonding due to the strong electron-withdrawing performance of fluorine atoms. The shared electron pair between fluorine atoms and carbon atoms shifts to the fluorine atoms greatly, which could provide a certain extent of protection for the negative charge layer of carbon atoms. It makes the fluorine-containing polymer present excellent weather resistance [25]. On the other hand, the extremely low polarizability of C–F bonding can reduce the intermolecular force between the air and the polymer surface [26], resulting in an extremely low surface energy and an excellent antifouling effect [27,28].

Catalytic chain transfer polymerization (CCTP) is an effective method and is a very efficient and versatile free-radical polymerization technique for the synthesis of macromonomers with u-unsaturated vinyl terminal groups [29]. McEwan et al. [30] obtained a range of branched polymers with an array of peripheral functionalities by CCTP and thio-Michael addition. Patias et al. [31] used the free-radical copolymerization of ω-unsaturated methacrylic macromonomers, as derived from CCTP, with acrylic monomers in solution leading to block copolymers by varying the nature of the macromonomer. Slavin et al. [32] synthesized copolymers of oligo(ethylene glycol) methyl ether methacrylates and allyl methacrylate by (CCTP) and reacted them with a-keratin in the human hair via Michael addition to cysteine residues.

To improve the comfortability of fabric with excellent antifouling performance, it is an important issue that should be solved in the textile field by reducing the adsorption and growth of microorganisms on the surface of the fabric. The betaine copolymer for the cotton fabric was synthesized by CCTP with the betaine monomer N,N-dimethyl (methacryloyloxyethyl) (DMAPS) and the fluorine-containing acrylate monomer tridecafluorooctyl methacrylate (G06B), and the silicone-oxygen coupling agent KH-590 was applied to enhance the washing fastness of cotton fabric after the betaine copolymer treatment. The contents of DMAPS and G06B on the temperature-sensitive performance and anti-protein specific absorption were investigated by the static water contact angle and an ultraviolet-visible spectrophotometer, respectively. This work will provide a basis for the construction of coating materials with excellent temperature-sensitive and anti-protein adsorption properties.

## 2. Materials and Methods

### 2.1. Materials

3-(2-Methacryloxyethyldimethylamino) propanesulfonate (DMAPS), bovine serum albumin (BSA), bis (difluoroboryl) dimethylglyoximato cobalt (CoBF), and fluorescein isothiocyanate (FITC) were purchased from Shanghai Aladdin Biochemical Technology Co., Ltd., Shanghai, China. Tridecafluorooctyl methacrylate (G06B) and azobisisobutyronitrile (AIBN) were supplied by Harbin Xuejia Fluorosilicon Chemical Co., Ltd., Harbin, China. Trifluoroethanol (TFE), phosphate-buffered solution (PBS), and γ-mercaptopropyltrimethoxysilane (KH-590) were ordered from Nanjing Quanxi Chemical Co., Ltd., Nanjing, China. Cotton fabric was provided by Shaoxing Keqiao Qianquan Textile Co., Ltd., Shaoxing, China, weighing 62.5 g/m^2^.

### 2.2. Manufacture of Betaine Copolymer

**Figure 1:**
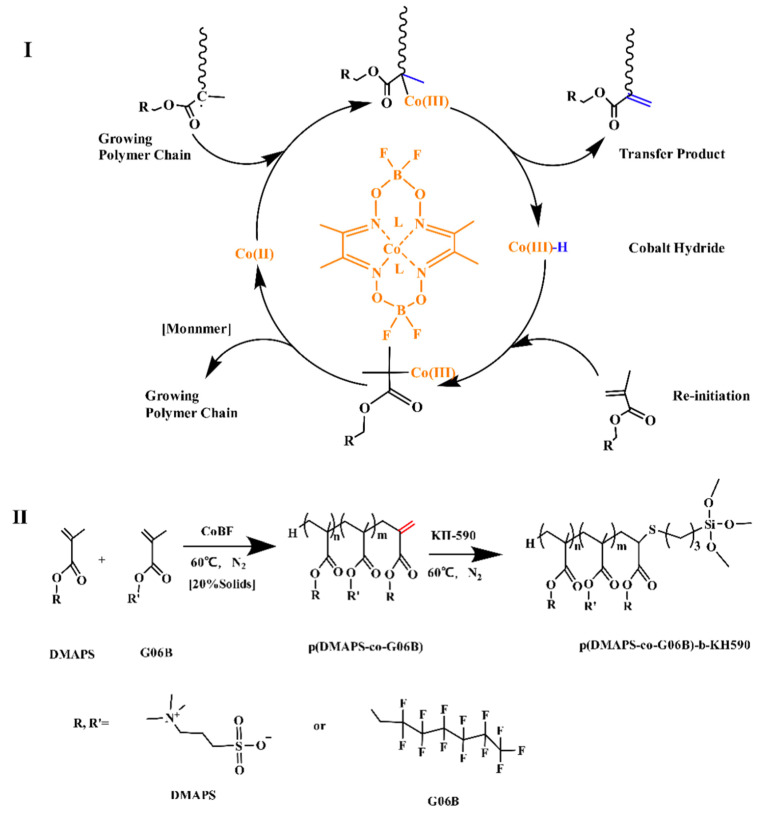
Synthesis diagram and flowchart of the synthesis process of betaine copolymer: (**I**) synthesis process; (**II**) synthesis diagram via CCTP.

The flowchart showing the synthesis process of the betaine copolymer is illustrated in Figure 1I, while the synthesis diagram via catalytic chain transfer polymerization (CCTP) is shown in Figure 1II. The first step is to use CoBF as the chain transfer catalyst, AIBN (used after recrystallization) as the initiator, trifluoroethanol (TFE) as the solvent, and DMAPS and G06B as the copolymerization monomers. The reactants were dissolved in a single-necked flask and sealed, N_2_ gas was blown for 0.5 h, and they were then reacted in a 60 °C water bath for 12 h to obtain p(DMAPS-co-G06B). The reaction was continued at 60 °C for 12 h with the addition of AIBN and KH-590, and finally, it was extracted with ethanol precipitation 3 times and then vacuum-dried at 60 °C for 36 h to obtain p(DMAPS-co-G06B)-b-KH590. The contents of monomer DMAPS, CoBF, and G06B for the betaine polymer copolymerization is recorded in Table 1.

### 2.3. Cotton Fabric Treatment

The cotton fabric was washed with ethanol to remove contaminates, and it was cut into several pieces with a dimension of 2 cm × 4 cm after drying at 60 °C. The concentration of betaine copolymer solution was 20 mg/mL, and it was then used to impregnate cotton fabric.

### 2.4. Methods

#### 2.4.1. Characterization of Betaine Copolymer

The proton nuclear magnetic resonance spectrum of the copolymer was determined by the Avance AV 400 MHz Fourier digital nuclear magnetic resonance spectrometer (^1^H–NMR, Bruker, Germany) at room temperature. The polymer specimen was prepared using a solvent of D_2_O.

The functional group of the betaine copolymer was characterized using the Vertex 70 spectrometer via Fourier Transform Infrared Spectroscopy (FTIR, Bruker, Germany) at an attenuated total reflectance mechanism. The scanning range was from 4000 cm^−1^ to 400 cm^−1^ with a resolution of 4 cm^−1^ and 32 times scanning.

#### 2.4.2. Surface Morphology Characterization of Cotton Fabric

The surface morphology of cotton fabric after treatment was observed using a scanning electron microscope (JSM-5610LV, JEOL Ltd., Akishima, Japan) with a working distance of 6 mm and high voltage of 30 kV.

#### 2.4.3. Temperature-Sensitive Performance Analysis of Cotton Fabric

To investigate the effect of the betaine copolymer on the temperature-sensitive properties of the cotton fabric, the static water contact angle of cotton fabrics in a continuous temperature-varying environment was characterized using a digital contact angle analyzer (DSA20, Kruss, Germany).

#### 2.4.4. Anti-Protein Adsorption Performance Analysis of Cotton Fabric

The cotton fabric with or without betaine copolymer treatment was washed with ethanol and deionized water, was placed into a sterilized petri dish with PBS solution for half an hour of immersion, and was then fully dried. After that, the BSA/PBS solution with a 2 mg/mL concentration was prepared for the cotton fabrics to immerse and incubate for 24 h to reach adsorption equilibrium. The BSA/PBS solution was taken out in the petri dish and an ultraviolet-visible spectrophotometer was used to measure the absorbance of the BSA/PBS solution under different adsorption conditions. Then, the modification was calculated based on Beer–Lambert law and the standard adsorption curve of bovine serum protein was calculated according to Equation (1) [33]:

(1) $$M=\frac{V}{m}\times \left(C_{0}-C_{1}\right)$$

where $C_{0}$ (mg/mL) is the concentration of the solute in the BSA/PBS stock solution, $C_{0}$ (mg/mL) is the concentration of the solute in the BSA/PBS solution after the adsorption equilibrium has been reached, and V (L) is the volume of the BSA/PBS solution. *m* (g) is the mass of cotton fabric.

## 3. Results and Discussion

### 3.1. Characterization of Betaine Copolymer

#### 3.1.1. ^1^H–NMR of Copolymer

**Figure 2:**
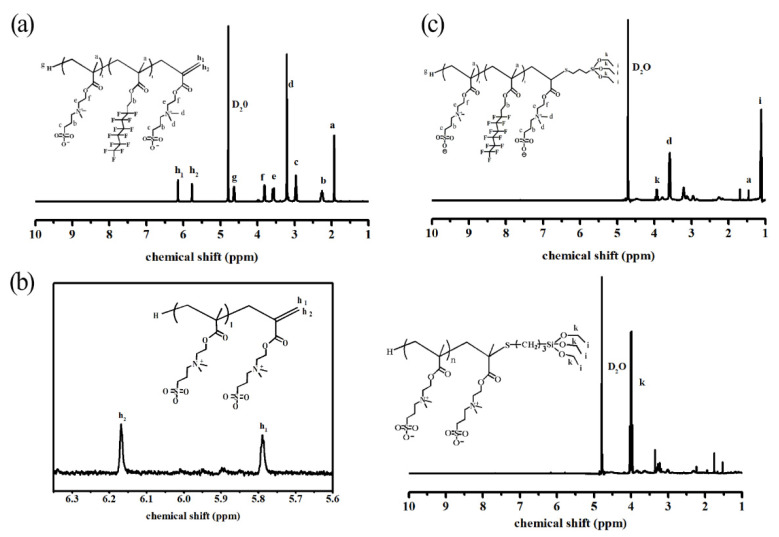
^1^H–NMR spectrum of temperature-responsive copolymer: (**a**) DMAPS + G06B copolymer (RUN 2); (**b**) DMAPS + 3 wt.%KH-590 copolymer (RUN 3); (**c**) DMAPS + G06B + 3 wt.%KH-590 copolymer (RUN 5).

It can be seen from Figure 2a that the ^1^H–NMR spectrum of the betaine copolymer containing the ω-vinyl group (δ = 6.16 ppm, δ = 5.75 ppm is the chemical shift of the vinyl proton peak) was similar to the predicted structural spectrum. This proves that the temperature-sensitive copolymer containing ω-vinyl at one end was successfully synthesized.

On the other hand, the ^1^NMR spectrum shown in Figure 2b presented a weak vinyl proton peak at shifts of 6.16 ppm and 5.75 ppm with the addition of 3 wt.% KH-590, indicating that the terminal vinyl copolymer did not react completely. However, the vinyl proton peak shown in Figure 2c completely disappeared with the addition of KH590 and G06B, whilst the –CH_3_ proton was found at the shift of 1.22 ppm, which indicated that the reaction with the vinyl-terminated copolymer could be completed.

#### 3.1.2. FITR Analysis of Copolymer

**Figure 3:**
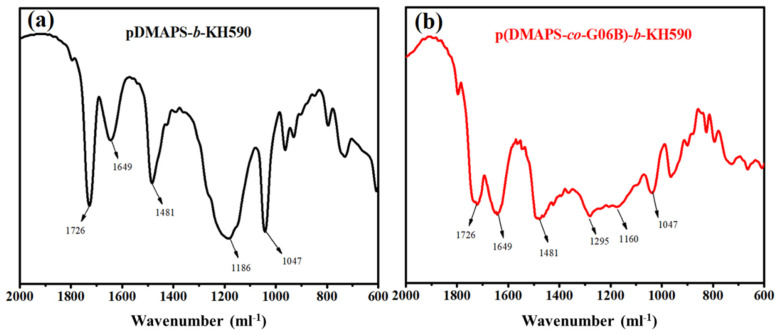
The FTIR spectrum of copolymer (**a**) PDMAPS + CoBF(40 ppm) (RUN 4); (**b**) PDMAPS + G06B + KH590 + CoBF(40 ppm) (RUN 5).

The functional groups of the copolymer before and after KH590 (3 wt.%) modification were characterized by FTIR, and the spectrum is shown in Figure 3. Based on the literature [6], the shifts of 1726 cm^−1^ and 1649 cm^−1^ were the characteristic absorption peaks of tridecafluoro methacrylate and C=O bonds, respectively, while the shift of 1481 cm^−1^ was the absorption peak of –CH_3_ of quaternary ammonium salt for betaine polymer (-N(CH_3_)_2_)-). In addition, the shift of 1186 cm^−1^ was the stretching vibration absorption peak for S=O in the anionic group sulfonate (–SO_3_) in the betaine polymer. According to Figure 3b, characteristic absorption peaks of C–F bonds were observed at the shifts of 1295 cm^−1^ and 1160 cm^−1^, which could be strong evidence to prove that tridecafluorooctyl methacrylate (G06B) took part in the reaction with betaine copolymer; the peak at 1090 cm^−1^ corresponded to the stretching vibration peak of the Si–O–Si bond, which indicated that the coupling agent KH590 was successfully grafted to the end of the copolymer. Thus, the betaine copolymer was successfully copolymerized with the fluorine-containing polymer and modified by the KH590 coupling agent.

### 3.2. Characterization of Cotton Fabric with Copolymer Coating

#### 3.2.1. FTIR-ATR Analysis of Cotton Fabric Surface

**Figure 4:**
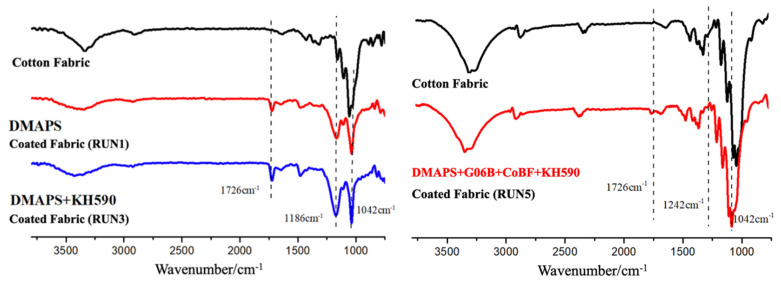
The FTIR spectrum of cotton fabric with the betaine copolymer-coating treatment.

FTIR-ATR was used to investigate the characteristic functional groups on cotton fabric before and after betaine copolymer treatment. FTIR-ATR spectra of control cotton fabric and cotton fabric with DMAPS + G06B + CoBF + KH590 (RUN 5) samples are presented in Figure 4.

The typical FTIR-ATR spectrum of the control presented broad absorption peaks, such as functional groups –OH (3600–3300 cm^−1^), –CH (3000–2800 cm^−1^), and –CO (1020 cm^−1^) bands. Additionally, for the FTIR-ATR spectrum of cotton fabric after the betaine copolymer treatment, the peaks at 1726 cm^−1^ and 1649 cm^−1^ were attributed to the stretching vibration absorption peak of C=O for tridecafluorooctyl methacrylate (G06B) and N, N-dimethyl (methacryloyloxyethyl) ammonium propane sulfonic acid inner salt (DMAPS), respectively. The peak of 1481 cm^−1^ corresponded to the asymmetric bending vibration absorption peak of –CH_3_ for the quaternary ammonium salt of the betaine polymer (–N(CH_3_)_2_–) structure, whilst the S=O stretching vibration absorption peak of the anionic group sulfonate (–SO_3_) for betaine polymer was found at the peak of 1186 cm^−1^, and the symmetrical stretching vibration peaks of C–O–C were recorded at 1042 cm^−1^ for the betaine polymer structure. As such, the structure of the betaine polymer on cotton fabric after finishing treatment could be confirmed.

Furthermore, the new characteristic absorption peaks of C–F bonds observed at the peaks of 1295 cm^−1^ and 1160 cm^−1^ could confirm the structure of fluorinated acrylate (G06B) on the cotton fabric, whilst the peak of 1042 cm^−1^ corresponded to the stretching vibration of the Si–O–C bond, which was evidence to prove that the structure of the silicon coupling agent KH-590 was grafted on the copolymer and coated on the cotton fabric. Therefore, it was confirmed that the betaine copolymer was successfully coated on the surface of the cotton fabric via finishing treatment.

#### 3.2.2. Surface Morphology Observation

**Figure 5:**
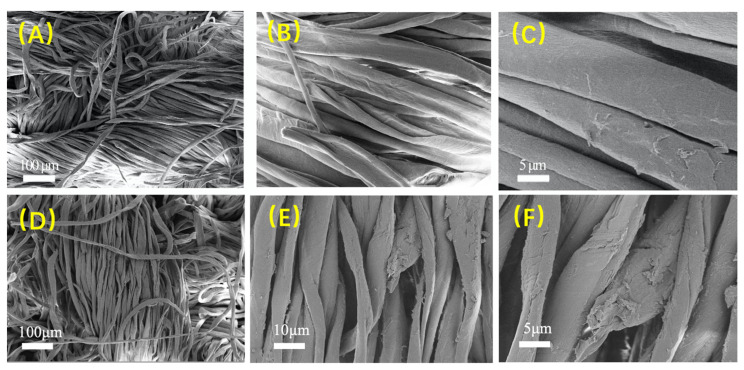
SEM images of cotton fabric with different magnifications: (**A**–**C**) Before finishing cotton fabric; (**D**–**F**) after p(DMAPS-co-G06B)-b-KH590 (RUN5) finishing treatment.

The SEM images of cotton fabric are illustrated in Figure 5, and the surface of unfinished cotton fabric fibers was relatively smooth and flat with clear boundaries between the fibers. However, a uniform copolymer film was observed on the fabric surface that was finished with the betaine copolymer, and gaps between fibers were filled with polymer. The polymer film was thin enough while the fibers of the cotton fiber became thinner, and the fibers of cotton fabric almost still retained the original pore structure. It could be predicted that the moisture absorption and perspiration performance of the cotton fabric would not be greatly affected after the betaine copolymer treatment.

#### 3.2.3. Temperature-Sensitive Performance Analysis

**Figure 6:**
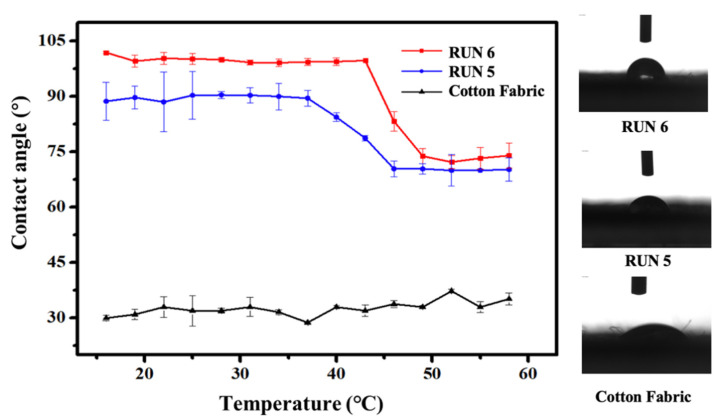
The curve of contact angle of water on fabric vs. continuous temperature.

The temperature-sensitive performance of cotton fabric was characterized by measuring the static water contact angle at continuous temperature. It can be seen from Figure 6 that the static water contact angle of the pure cotton fabric was around 30° at different temperatures.

After finishing treatment with betaine copolymer with p(DMAPS-co-G06B)-b-KH590 (RUN 5), the static water contact angle of the modified cotton fabric obtained was about 85° when the temperature was below 46 °C, which presented a better hydrophobicity compared with raw cotton fabric. The contact angle of the coated cotton fabric suddenly reduced to 75.1° when the temperature increased to 46 °C, indicating the hydrophilic transformation with a certain temperature sensitivity. However, no continuous decrease was observed for contact angle with further increases in temperature. 

In addition, the static water contact angle of the modified cotton fabric at 46 °C was increased to around 100° with the increase in the contents of G06B in p(DMAPS-co-G06B)-b-KH590 (RUN6). As such, the hydrophilic/hydrophobic transition temperature was increased slightly, and the static water contact angle after the transformation was approximately 76 °C.

It can be obtained from Figure 6 that the average contact angle of RUN 5 had a higher variation under 20–30 °C, due to the average contact angle being directly related to the upper critical solution temperature (UCST) of PDMAPS. If the temperature was lower than the UCST, the intramolecular hydrogen bonds in PDMAPS formed and the PDMAPS coating was hydrophobic. Otherwise, the PDMAPS molecular chain stretched and formed hydrogen bonds with water in the environment. The RUN 5 had a higher content of DMAPS, and the UCST of PDMAPS was around 20–30 °C, resulting in the average contact angle of RUN 5 having higher variation under 20–30 °C.

#### 3.2.4. Anti-Protein Specific Adsorption Performance

**Figure 7:**
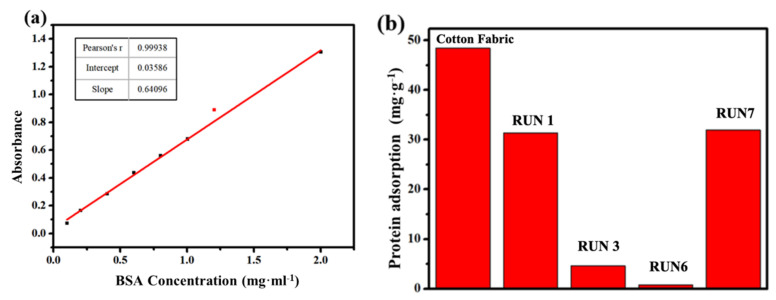
UV-visible spectrophotometer analysis: (**a**) protein (BSA) adsorption standard curve; (**b**) protein adsorption behavior of cotton fabric (mg/g).

A standard concentration gradient of BSA/PBS solution was prepared to test the absorbance at the maximum absorption wavelength with an ultraviolet-visible absorbance photometer to obtain the standard adsorption curve of BSA concentration and absorbance, as shown in Figure 7a. The initial concentration of the protein solution was 1.0 mg/mL, for the easy observing of the protein adsorption behavior of treated fabric, the BSA concentration was doubled, and the maximum range of 2.0 was set. If the maximum was smaller than 2.0, the measured protein adsorption behavior would not be good enough. Otherwise, the experimental error would be amplified, which would make the results unreliable.

The absorbance of the adsorbed BSA solution of different samples was measured, and the amount of bovine serum protein adsorbed per unit mass of cotton fabric was calculated and is shown in Figure 7b. According to Figure 7b, the pure betaine polymer (RUN1) also presented a certain anti-protein (BSA) adsorption performance. When the fluorine-containing monomer (G06B) was copolymerized with the betaine monomer, the anti-protein specific adsorption performance of the cotton fabric finished with copolymer was further improved. The modified cotton fabric (RUN6) presented the best anti-protein specific adsorption behavior with 0.8 mg/g of bovine serum albumin (BSA), which was approximately reduced by 98% when compared to cotton fabric without any treatment, and the anti-protein specific adsorption performance of RUN6 was only 0.96%. However, with the further increase in the content of G06B, the protein adsorption resistance of the modified cotton fabric was observed to be decreased (RUN7). This was due to the decrease in the content of betaine zwitterionic monomer, which resulted in a weakened hydration layer built on the cotton fabric surface with uneven distribution.

## 4. Conclusions

The betaine copolymer p(DMAPS-co-G06B)-b-KH590 was designed and synthesized by the catalytic chain transfer polymerization (CCTP) method, and the temperature sensitivity and specific anti-protein adsorption performance of the cotton fabric, which was modified by the betaine copolymer, was investigated. The following conclusions can be obtained:

1. The structure of p(DMAPS-co-G06B)-b-KH590 copolymer was confirmed by ^1^H–NMR and FTIR, and the betaine copolymer was successfully coated on the surface of the cotton fabric via finishing treatment. It was proven that moisture absorption of the cotton fabric would not be greatly affected by the betaine copolymer.
2. The betaine copolymer-treated cotton fabric exhibited a hydrophobicity/hydrophilic transition around 40 °C, which was hydrophobic at low temperature and hydrophilic at high temperature. In addition, the functional fabric could present a better anti-protein specific adsorption performance. The specimen RUN 6 had the best temperature-sensitive and anti-protein specific absorption performance among all the specimens.

## Figures and Tables

**Table 1:** The formulation of the betaine-type copolymer for characterization.

RUN	DMAPS (g)	G06B (g)	CoBF	KH590	AIBN (g)	TFE (g)
1	3.00	/	/	/	0.09	17
2	3.00	0.03	/	/	0.09	17
3	3.00	/	/	3 wt.%	0.09	17
4	3.00	/	40 ppm	3 wt.%	0.09	17
5	2.91	0.09	40 ppm	3 wt.%	0.09	17
6	2.82	0.18	40 ppm	3 wt.%	0.09	17
7	2.28	0.72	40 ppm	3 wt.%	0.09	17
